# Real-World Use of Generic Meropenem: Results of an Observational Study

**DOI:** 10.3390/antibiotics10010062

**Published:** 2021-01-11

**Authors:** Santiago Garnica-Velandia, Luz Adriana Aristizábal-Ruiz, Carlos Arturo Alvarez-Moreno

**Affiliations:** 1 Vitalis SACI, Bogota 111121, Colombia; adriana.aristizabal@vitalis.com.co; 2 Internal Medicine, Department Facultad de Medicina, Universidad Nacional de Colombia, Bogota 111176, Colombia; caalvarezmo@unal.edu.co; 3 Infectology Department, Clínica Universitaria Colombia, Clínicas Colsanitas S.A, Bogota 111176, Colombia

**Keywords:** antimicrobials, meropenem, generic drug, real-world studies, product surveillance, postmarketing, treatment outcome, pharmacovigilance

## Abstract

Background: To determine the therapeutic effect and tolerability of meropenem in routine clinical practice, in terms of clinical and microbiological response. Methods: A real-world, observational, descriptive, longitudinal study with daily monitoring of clinical history records was conducted on all patients who were medically prescribed meropenem during a period between October 2015 and March 2016 at a university hospital in Bucaramanga (Colombia). Results: The study evaluated 84 patients with an average age of 63.2 years, mostly older adults with multiple comorbidities, of whom 54.8% were men. A positive clinical or microbiological response was obtained in 98.8% of the patients. At the end of the treatments, significant improvements in dysthermia (0% vs. 29% at the beginning, *p* = 0.000), tachycardia (13% vs. 47%, *p* = 0.049), and leukocytosis (39% vs. 15% at the beginning, *p* = 0.008) were evidenced. The improvement in the indicator that combines all the Systemic Inflammatory Response Syndrome (SIRS) criteria was also significant (*p* = 0.000). The treatment was well tolerated, although we identified some non-serious and expected adverse reactions. Conclusions: Generic meropenem proved to be effective and well tolerated for different types of infection in routine clinical practice. The results are consistent with the findings of the clinical studies with the innovator drug.

## 1. Introduction

Antimicrobials have been of great therapeutic use for more than half a century as indispensable tools for treating a wide variety of infections [1,2], enabling to save the lives of millions of people. Meropenem is a widely used antimicrobial in the treatment of many complicated infections. Its wide spectrum, its powerful effect on microorganisms with different resistance mechanisms, and its good tissue penetration make it a highly useful part of the therapeutic arsenal [3,4,5]. As such, it is important to monitor its use and results at a local level in order to conserve its effectiveness and its safety in terms of development of bacterial resistance and other related problems. In this sense, real-world studies are becoming increasingly important as a trustworthy source of evidence for the behavior of treatments in routine clinical practice and as a support for clinical decision judgment. Furthermore, these studies include patients that are normally excluded from clinical studies and provide information about the local use of medications, allowing us to identify factors that can have an impact not only regarding safety but also in terms of therapeutic results [6,7].

Optimizing health care expenditures is a priority for the governments of many countries, and the introduction of generic drugs to the market dramatically lowers health care costs while facilitating access to medicines. The effectiveness of different generic antimicrobials has previously been studied in different situations [8,9,10]. At a local level, there is evidence for its equivalence to trade medications in terms of mortality, length of hospital stay, and safety [11]. Studies from around the world have described positive results for meropenem in terms of its effectiveness and tolerability both in complicated infections and in other important conditions [12,13,14,15]. However, local evidence is limited, and there is a need for real-world studies that can support decision making, even more when some doctors and patients do not completely trust generic drugs. In this sense, observational studies in real life are important, which allow evaluating the therapeutic effect of generic drugs in a context of routine use once they have been approved. This study sought to address this need by determining the effect of this carbapenem in terms of the clinical and microbiological response in patients treated in a six-month period at a university hospital in Colombia.

## 2. Results

**Figure 1:**
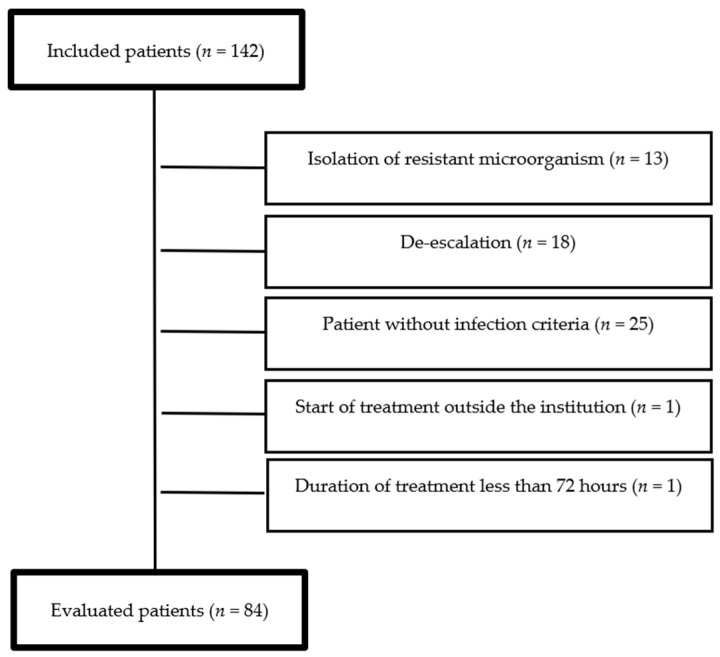
Study flow diagram.

A total of 142 meropenem treatments were included, of which 58 (40.8%) met the above-mentioned exclusion criteria (Figure 1). Thus, 84 patients were finally evaluated. The average age was 63.2 years (SD = 19). The average body mass index (BMI) was 23.5 (SD = 3.2). The average length of stay was 18 days (SD = 11). The main comorbidities, types of infection, and etiological agents are described in Table 1.

The etiological agent was identified by culture in two-thirds of the patients (*n* = 56, 66.6%). The most frequently isolated microorganism was *Pseudomonas aeruginosa* (*n* = 18, 32.1%), followed by *Escherichia coli* (*n* = 16, 28.6%) and *Klebsiella pneumoniae* (*n* = 10, 17.9%). Nearly a half of isolated bacteria were producers of extended spectrum beta-lactamases (ESBL) (*n* = 25, 44.6%). We found resistance to third-generation cephalosporins in 24 cases (42.8%), resistance to aminoglycosides in 36 cases (64.3%), and resistance to quinolones in 30 cases (53.6%).

In most patients, the dose used was 1 gram every 8 hours (*n* = 72, 85.7%). Other prescription regimens are described in Table 2. The specialty that most prescribed meropenem was internal medicine (*n* = 45, 53.6%). The median treatment duration was 9 days (interquartile range 7-12).

At the end of meropenem treatment, the effect was measured in each Systemic Inflammatory Response Syndrome (SIRS) criteria individually. Qualitatively, the response was significant for dysthermia, tachycardia, and leukocytosis (Table 3), and quantitatively in the four variables: body temperature (*p* = 0.000), breathing rate (*p* = 0.018), heart rate (*p* = 0.000), and leukocyte count (*p* = 0.000).

**Figure 2:**
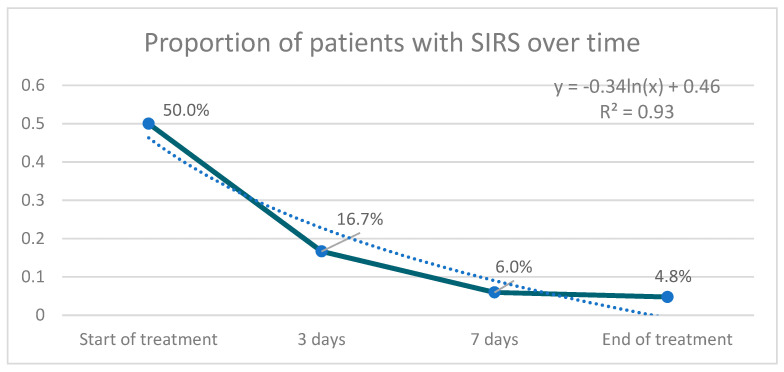
Proportion of patients with SIRS (Systemic Inflammatory Response Syndrome) over time treatment.

The proportion of patients with SIRS at the end of treatment was significantly lower than at the beginning (*p* = 0.05) (Figure 2). The aggregate response of the SIRS criteria through the combined indicator was also significant (*p* = 0.000 for difference of means, *p* = 0.000 for difference of variance).

The therapeutic objective was achieved in 83 of the 84 treatments analyzed (98.8%). Most of the patients presented clinical response (*n* = 82, 97.6%), while complete response was evident in 23.8% of the cases (Table 4). During the monitoring, we identified some patients who presented non-serious and expected adverse reactions, such as skin manifestations of hypersensitivity (*n* = 6, 7.1%), constipation (*n* = 4, 4.8%), and a patient with oral candidiasis (*n* = 1, 1.2%). We found no clinical or paraclinical evidence of potential interactions of meropenem with other drugs.

## 3. Discussion

Different clinical studies with meropenem show efficacy between 64% and 99%, depending on the type of infection. The highest success rate has been achieved in urinary tract infections (90.0–99.0%), followed by skin and soft tissue infections (73.1–98.0%) and lower respiratory tract infections (64.0–89.0%) [3,4]. Meanwhile, real-world studies have shown that the effectiveness of generic meropenem has ranged from 72.5% to 80.0% [12,13,14,15]. In the present study, the effectiveness was determined to be 98.8%, which is within the range reported in clinical studies and is higher than in other real-world studies. This may be because patients with inadequate therapy were not included in this study, i.e., those cases where non-meropenem-susceptible microorganisms were identified.

The therapeutic effect of meropenem in this study was measured in terms of clinical response and microbiological clearance. Clinical response was determined through judgment of the treating physicians and the improvement of SIRS. Qualitative and quantitative methods were used to compare treatment effect both for each SIRS criterion individually and for all of them together. The literature reports evidence about the significantly superior performance of the SIRS criteria for diagnosing sepsis in patients outside the Intensive Care Unit as is the case of this study [16]. Improvement of SIRS throughout treatment can also be considered as an indicator of clinical response in infected patients with SIRS and a microbiological isolate. This study found the disappearance of SIRS over time for a significant proportion of patients, despite complex factors that threaten therapeutic success, including older patients with multiple comorbidities such as hypertension, diabetes, chronic obstructive pulmonary disease, kidney disease, and infection with human immunodeficiency virus. These baseline conditions are associated with various complications, including increased hospital stay and mortality in respiratory infections [17,18,19], multi-resistance in urinary pathogens [20,21,22], and worse prognosis in bacteremia [23].

The case of therapeutic failure corresponds to an 82-year-old patient with congestive heart failure, stage III chronic renal disease, hypothyroidism, and COPD, who was treated in the intensive care unit for cardiogenic shock, atrial fibrillation, and respiratory failure requiring invasive mechanical ventilatory support. In addition, there was suspected myelodysplastic syndrome due to bicytopenia, which progressed to pancytopenia. The patient presented broncho-aspiration pneumonia, for which he received meropenem for 10 days. He went on to suffer from dyspnea, tachycardia, subsequent multiorgan failure, and finally died. The expected mortality according to the Acute Physiology And Chronic Health Evaluation II (APACHE II) was 40%, rising to 80% due to comorbidities, despite optimal treatment. At the end of the treatment with meropenem, *Stenotrophomona maltophilia* was isolated in tracheal aspirate. This bacterium is an opportunistic microorganism that, as it possesses intrinsic resistance to several antimicrobials, could be associated with the outcome. It is inducible for L1 beta-lactamases with class B zinc enzymes, highly active against carbapenems, and by protocol, it is only tested by antibiogram against trimetroprim/sulfamethoxazole, which is the treatment of choice [24].

Cultures were taken in only two-thirds of the patients, which could be due to the difficulty of obtaining samples in cases of pneumonia. A second culture was taken to assess for sterility and/or clearance of the bacteria after the meropenem treatment in less than half of the cases with previous positive bacterial cultures. Regarding the isolated microorganisms, we mainly identified pathogens that have developed significant antimicrobial resistance: *Pseudomonas aeruginosa*, *Escherichia coli,* and *Klebsiella pneumoniae*.

The most frequently used dosage was 1 g every 8 h, which is in accordance with the dosage recommendations established for this carbapenem. Dose adjustments were identified at the end of treatment in less than half of the patients with renal disease (*n* = 12, 42.9%), particularly to regimens of 500 mg every 12 h and 1 g every 24 h. This proportion is considered low, and configures as a factor to be improved toward antimicrobial safety. On the other hand, some cases of bacteria with increased minimum inhibitory concentration required adjustment to doses of 2 g every 8 h. In three cases, the treatment lasted more than 40 days, two of which corresponded to osteoarticular infections and another corresponded to a case of perianal abscesses that were difficult to treat. Regarding concurrent antimicrobials, 10 patients with co-infection required vancomycin to increase coverage against Gram-positive bacteria.

As an important contribution regarding the rational use of meropenem, in 18 patients who received initial empirical treatment with meropenem, the treating physicians decided to de-escalate based on the result of the antibiogram. The mean duration of meropenem therapy prior to de-escalation was 3.8 days (S.D. = 0.8), which is time that can be improved in terms of variation of bacterial susceptibilities in Colombia, by promoting quick tests and stewardship protocols absent in the hospital at the time of this study. The use of meropenem as initial therapy in the emergency department by general practitioners is also noteworthy; however, we confirmed that these were professionals trained by the urology department who started the treatment following the criteria established by this institution.

Around the world, it has become evident that there is a need to strengthen medical education by avoiding the prescription of antimicrobials in cases that do not warrant it. In this study, we identified cases without indication for antimicrobial use, including asymptomatic bacteriuria (ASB) and colonization (*n* = 17, 12.0%) [25]. Another issue to highlight is that no dosing adjustments were seen in eight hemodialyzed patients, bearing in mind that up to 50% of meropenem is removed during this process, and so a dose is required after each session [26,27]. These considerations for usual practice can be modified in order to prevent the development of bacterial resistance, in this case carbapenemase production.

In terms of safety and tolerability, the adverse reactions identified were non-serious, expected, and their frequency is within the expected range [4,5]. Some analyses indicate that cross-reactivity between penicillin and meropenem is low (0.06–1.9%); however, vigilance and caution are recommended [28]. Two of the seven patients with previous hypersensitivity to penicillins presented cutaneous manifestations. The first one was given a dose of piperacillin/tazobactam before increasing the dose of meropenem, and the other patient received six concomitant drugs, which makes the association with meropenem less likely, but they are still plausible cases. Another possible adverse event of special interest in terms of safety is the lowering of the seizure threshold when using carbapenems [5]. Despite observing patients with risk factors, this potential reaction was not identified in any case.

In the context of decision-making and efficiency in health expenditure, the World Health Organization has urged developing countries to promote the use of quality generic medicines. Some animal models have suggested that the behavior of the generic drug is not equivalent to that of the innovator [29]; however, they have been questioned, as they have not been validated to assess antimicrobial efficacy [8]. This study shows generic meropenem to be effective and well tolerated for different types of infection in routine clinical practice.

The strengths of this study include the precision in obtaining the data, the frequent monitoring, having considered 100% of the patients eligible (thereby guaranteeing representativeness in those risk groups that are usually excluded from clinical studies) and not considering those cases in which there is no evidence of infection, even though they could have been considered successful examples of bacterial eradication in cases such as asymptomatic bacteriuria. The limitations include the small number of patients, implementation at a single hospital center, and non-inclusion of outcomes such as hospital stay and mortality.

In future research, it is recommended to evaluate compliance with institutional antibiotic use guidelines and the ways the medication is used by the nursing group, including the reconstitution and dilution technique, infusion time, and compliance with the timing of antimicrobial administration. Likewise, it is important to make an in-depth evaluation of pharmacokinetic aspects of interest such as drug interactions and renal failure, with their respective dose adjustments, correlating these variables with the outcomes. 

## 4. Materials and Methods 

We carried out a real-life, descriptive, longitudinal, and prospective observational study at a tertiary university hospital in the city of Bucaramanga (Colombia), through the daily monitoring of all the consecutive patients who were medically prescribed meropenem in a six-month period between October 2015 and March 2016. 

We included patients of all ages, degrees of severity, comorbidities, and types of infection who were given meropenem (Vitalis, Colombia) regardless of the services and speciality of the institution that referred them. In order to characterize the patients, we collected demographic, clinical, laboratory, pharmacological, and microbiological variables based on their clinical history records in a case report form (CRF). The information was collected and collated by a trained professional in a previously tested and adjusted database (Microsoft Excel). The data obtained were validated by the researchers.

The following were considered as exclusion criteria: (a) non-compliance with the criteria defined in the reference literature to confirm infection, such as bacteremia [30], intra-abdominal infections [31], skin and soft tissue infections [32], diabetic foot [33], urinary tract infections [34,35], community-acquired pneumonia [36], and healthcare-associated pneumonia [37], (b) if the microorganism causing the infection was identified as being resistant to or not susceptible to meropenem, (c) de-escalation based on an antibiogram for meropenem, (d) the patient was treated for less than 72 h, and (e) if the treatment began in another institution.

Effectiveness was defined as the frequency of cases that had a clinical response, microbiological clearance, or both. Cases that had both clinical and microbiological improvement were classified as having a complete response. Clinical response was defined as (a) explicit evidence of resolution or improvement of clinical symptoms and signs of infection, in the judgment of the treating physicians, or (b) evidence of disappearance of SIRS following treatment [38,39]. In applicable cases, the treatment includes surgery in order to control the source of the problem (e.g., infected diabetic foot). Microbiological response was defined as a one successive negative culture from the same site as from where the Gram-negative bacteria was originally isolated. Therapeutic failure corresponded to those cases in which there was no clinical or microbiological response [40].

In order to identify potential interactions between meropenem and the other concomitant medications, a list of possible events of clinical relevance was created, by consulting a validated online tool for this purpose [41].

The data were analyzed using the free software R (The R Foundation). Results were expressed by univariate analysis in percentage frequencies, means with standard deviations, and medians with interquartile ranges. Bivariate analyses were performed to study the change in each SIRS criteria between the beginning and end of treatment and likewise the change in the SIRS as a whole. In qualitative analyses (yes/no), we used chi-square and Fisher’s exact tests, and for quantitative analyses, we used the t-test for difference of means or the Wilcoxon test for non-normal distributions. Additionally, a unified indicator was created by means of standardization to evaluate the response of all the SIRS variables before and after the treatment. In all cases, the level of statistical significance was set as *p* ≤ 0.05.

## 5. Conclusions 

With these findings, it can be concluded that treatments with meropenem (Vitalis) in the usual clinical practice proved to be effective and well tolerated, with results that are within those expected according to previous data in clinical and real-world studies. As regards opportunities for improvement in the use of meropenem, several factors were identified that can be managed to mitigate the risk of failure and generation of bacterial resistance.

## Figures and Tables

**Table 1:** Characteristics of the patients (*n* = 84).

Variable	*n*	%
Age		
18–65	39	46.4
≥65	45	53.6
Sex		
Female	38	45.2
Male	46	54.8
Comorbidities		
Hypertension	75	89.2
Diabetes mellitus	42	50.0
Chronic obstructive pulmonary disease	28	33.3
Renal disease	28	33.3
Cancer	19	22.6
Malnutrition	19	22.6
HIV infection	4	4.7
Indication		
Urinary tract infection	21	25.0
Pneumonia	17	20.2
Skin and soft tissue infection	13	15.5
Intra-abdominal infection	10	11.9
Operative site infection	6	7.1
Primary bacteremia	5	6.0
Another type of infection *	12	14.3
Etiological agent		
*Pseudomonas aeruginosa*	18	32.1
*Escherichia coli*	16	28.6
*Klebsiella pneumoniae*	10	17.9
Another etiological agent **	12	21.4

* diabetic foot, infective exacerbation of Chronic Obstructive Pulmonary Disease, tracheobronchitis, septic arthritis, empyema, pelvic infection. ** Enterobacter cloacae, Klebsiella oxytoca, Citrobacter freundii, Morganella morganii, Citrobacter spp, and Pantoea agglomerans.

**Table 2:** Characterization of the prescription of meropenem (*n* = 84).

Variable	*n*	%
Dose		
1 g every 8 h	72	85.7
1 g every 12 h	5	6.0
2 g every 8 h	3	3.6
0.5 g every 12 h	2	2.4
1 g every 24 h	2	2.4
Duration of treatment		
4–6 days	10	11.9
7 days	23	27.4
8–13 days	32	38.1
14 days	14	16.7
>14 days	5	5.9
Prescriber specialty		
Internal Medicine	45	53.6
Infectology	9	10.7
Urology	8	9.5
Intensive care	8	9.5
Others	14	16.8

**Table 3:** Qualitative individual response in the Systemic Inflammatory Response Syndrome (SIRS) criteria.

Variable	*n* (%)	*n* (%)	*p* Value
Dysthermia	29 (34.5)	0 (0.0)	0.000
Tachycardia	47 (56.0)	13 (15.5)	0.050
Tachypnea	27 (32.1)	7 (8.3)	0.832
Leukocytosis	39 (46.4)	15 (17.9)	0.008

**Table 4:** Achievement of therapeutic objective with meropenem (*n* = 84).

Response Type	*n*	%
Complete response (clinical + microbiological)	20	23.8
Clinical response	62	73.8
Microbiological response	1	1.2
Total responding patients	83	98.8

## Data Availability

Restrictions apply to the availability of these data. Data was obtained from “Los Comuneros” University Hospital. The data presented in this study are available on request from the corresponding author.
